# “Christian Science” Cures

**Published:** 1889-08

**Authors:** 


### "CHRISTIAN SCIENCE" CURES.

Dr. Pepper, of Philadelphia, in one of his clinical lectures—subject, a case of nervous dyspepsia—said :

" There is a state of fixed attention to the suffering part always present in this class of cases which often continues after the disease is cured. It is the recognition of this condition which forms the basis of what is known as the 'faith cure,' a therapeutical agent which has, gentlemen, claims on our most serious study, and I will go so far as to say that without 'faith cure' you cannot treat cases of nervous dyspepsia with success. I have seen cases which have been for months under the care of intelligent physicians, to be completely cured after two or three interviews with the faith healer, who says, ' There is no such thing as disease, therefore you are not sick, you are not suffering, you must not suffer.' This is a line of argument which, in the hands of skilful physicians, is a powerful remedial agent, but when used by unscrupulous persons degenerates into the merest quackery. By its means you obtain the confidence of your patients, you get their attention removed from the affected part, and you break up the habit. You cannot over-estimate the importance of the emotional element in these cases ; but if you are content with removing this alone, you are merely the most superficial empiric."

